# Modeling differences in neurodevelopmental maturity of the reading network using support vector regression on functional connectivity data

**DOI:** 10.1016/j.dcn.2026.101716

**Published:** 2026-03-25

**Authors:** Oliver H.M. Lasnick, Jie Luo, Brianna Kinnie, Shaan Kamal, Spencer Low, Natasza Marrouch, Fumiko Hoeft

**Affiliations:** a University of Connecticut, Storrs-Mansfield, CT 06269, USA; b Stanford University School of Medicine, Stanford, CA 94305, USA; c University of Connecticut School of Medicine, Farmington, CT 06030, USA; d Mass General Hospital, Boston, MA 02114, USA; e University of Lisbon, Lisbon 1649, Portugal

**Keywords:** Brain-age, Support vector regression (SVR), Maturation, Dyslexia

## Abstract

Predicting chronological age from functional properties of the brain may be informative of children’s neurodevelopmental trajectories. Specifically, neurodevelopmental or learning disorders may be associated with atypical maturation of brain networks. In this study, we examine the relationship between reading disorder (RD) and maturation of functional connectivity (FC) in the brain using a cross-sectional sample of N = 742 participants aged 6–21 years. A support vector regression model was trained to predict chronological age from FC data derived from a whole-brain model as well as multiple ‘reduced’ models, which were trained on FC data generated from a successively smaller number of regions in the brain’s reading network. We hypothesized that the trained models would show systematic underestimation of brain network maturity for poor readers, particularly for the models trained with reading/language regions. Results showed that the most highly weighted regions/functional connections were derived from the default mode and frontoparietal control networks, in line with previous research. The trained whole-brain model implied that participants with RD have a larger brain-age gap (difference between real and predicted age) than controls with typical or advanced reading ability; however additional post-hoc testing only confirmed a tendency for relative *over*estimation of age in advanced readers in the older cohort. Overall, results suggest that while FC networks may be particularly susceptible to, or reflective of, variation/developmental deviation associated with reading ability, there is large individual variability in the examined population.

## Highlights

• A machine learning model trained on functional data predicted participants’ ages
• The model showed variability in age prediction accuracy based on reading skills
• The model highly weighted data from frontoparietal and default mode regions
• Neural markers of reading and language are diffusely represented in the brain

## Graphical abstract

**Figure:**
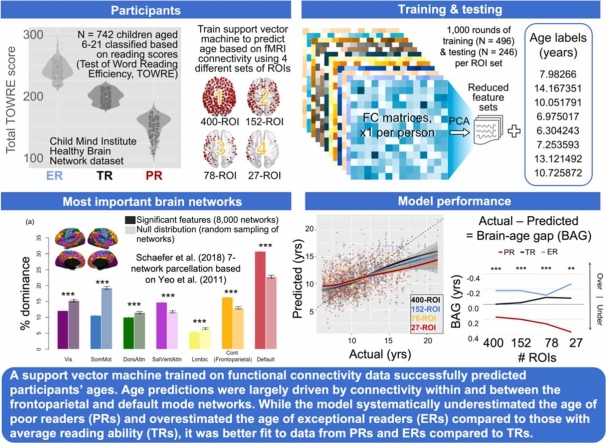

## 1 Introduction

Neuroanatomical and functional properties of the brain undergo key developmental changes from infancy all the way until early adulthood (Stiles and Jernigan, 2010). Tracking typical and atypical trajectories of brain development is crucial for understanding the progression of neurodevelopmental disorders (Wang et al., 2021). Researchers have previously tracked the maturation of brain structure and function from childhood to adulthood (Fair et al., 2007, Fair et al., 2008, Dong et al., 2020). Regions in the default mode network (DMN), a network that is most active during wakeful rest and posited to play diverse roles in social cognition, memory, and multimodal integration (Smallwood et al., 2021), shift from being sparsely interconnected to densely interconnected with increasing age (Buckner et al., 2008, Fair et al., 2008). In general, short-range connections tend to be negatively correlated with age (decrease in strength from childhood to adulthood), while long-range connections grow stronger with age (Fair et al., 2007).

A key area of this research employs machine learning models to predict ‘brain age’, or neural network maturation, from brain data. For instance, Dosenbach et al. (2010) used support vector regression (SVR) to train a model to predict brain age/maturity from functional connectivity (FC) data. A model that can reliably predict age from brain data in normative populations may be useful in the detection of developmental deviations within clinical populations (for an example in those with attention deficit hyperactivity disorder, see Kessler et al., 2016). If a child has altered early brain development, their estimated brain age could differ substantially from their chronological age, which is reflected as a larger brain age gap (BAG, An et al., 2025).

One area where the brain-age estimation remains underexplored is in developmental dyslexia, or specific reading disorder (RD) (Peterson and Pennington, 2015, Swagerman et al., 2017). RD is characterized by difficulties in reading that cannot be explained by intellectual disability or sensory perception deficits. Deviations in brain age for RDs compared to their same-age, typically developing peers may indicate atypical maturation in neural systems essential for reading. A younger estimated brain age relative to true age could suggest the brain has not reached the biological stage needed for fluent reading, potentially explaining the persistent difficulties seen in children with RD despite adequate instruction. Importantly, brain age estimates can be derived from either whole-brain data (traditional approach) or from distinct sets of brain regions. Isolated underdevelopment of reading-related brain regions that does not extend to other brain regions could explain the specific reading and language deficits observed in RD. Reading recruits widely distributed left-hemisphere ventral occipitotemporal, inferior frontal, and posterior parietal regions in both children and adults, and those with RD have reduced functional activity in inferior parietal cortex, the superior temporal gyrus, and the inferior frontal gyrus (Maisog et al., 2008, Martin et al., 2015, Bailey et al., 2018). Many of these regions are part of the frontoparietal attention network and the DMN (Bailey et al., 2018); therefore, models trained to predict age from data in these regions may also capture brain features associated with reading ability.

To the best of our knowledge, no studies to date have aimed to compare the brain-age gap between high-, mid-, and low-level readers. Brain-age gap estimation offers a theoretical framework to distinguish whether RD reflects a maturational lag that is brain-wide or isolated to specific reading-related regions. Such markers may one day complement behavioral screening for early RD risk detection.

Our overarching goal was to construct a model that successfully predicts age in a cross-sectional sample of children and adolescents with a wide range of reading ability. We included a wide age range to ensure the model was sufficiently trained on brain development up to early adulthood, which is still a highly-active period of brain development (Arain et al., 2013). For our model we chose to use linear SVR, a well-known supervised learning algorithm that predicts continuous outcomes by finding the best-fit function within a defined error margin (Drucker et al., 1997). SVR provides robust support for training using high-dimensional neuroimaging data with limited dataset size: it is resilient to outliers and can produce reliable performance even with relatively small training samples (Kumari and Sundarrajan, 2024). Additionally, researchers have previously used SVR to construct brain age-prediction models (Dosenbach et al., 2010, Erus et al., 2015, Kumari and Sundarrajan, 2024).

Our SVR model was trained to predict age from FC data collected during a naturalistic audiovisual (movie-watching) paradigm (see Materials and Methods). While prior work has shown that the architecture of whole-brain networks derived from resting-state and task fMRI are highly similar (Cole et al., 2014), and that rs-fMRI affords greater flexibility for investigating large-scale network maturation and for examining post hoc brain-behavior associations across development (Uddin and Garavan, 2025), naturalistic movie-watching may have additional advantageous properties: there is some evidence that these paradigms produce functional data with higher reliability than intrinsic resting-state or task-based fMRI (Wang et al., 2017, Aliko et al., 2020).

First, we assessed the significance of our age prediction model and compared the predictive ability of models trained with varying sets of FC data (see Materials and Methods).

Second, we conducted exploratory analyses aimed at determining which brain regions and connections drive model performance. To achieve this goal, we assessed the relative importance of each functional connection based on model weights.

Finally, we examined whether our trained model is able to distinguish the developmental trajectories of exceptional, typical, and impaired readers. To this end, our sample also includes precocious readers (children with unexpectedly advanced reading skills for their age or IQ; Ostrolenk et al., 2017), with the expectation that this sample might also show altered patterns of development in comparison to the typical-range and impaired readers. In line with a proposed “maturational lag” hypothesis of RD, we hypothesized that our SVR model would systematically underestimate the age of participants with lower reading ability, quantified as a larger positive BAG (Satz et al., 1978, Black et al., 2017). An important methodological aspect of this analysis is our decision to create multiple models trained on successively smaller number of functional connections: we construct models trained with data from either whole-brain or reading-specific brain regions and investigate whether the magnitude of the group effect on the BAG differs based on participants’ reading ability. We hypothesized that the group effect on the BAG would be larger for the more reading-specific models, trained with data from a limited number of ROIs, compared to the whole-brain model.

## 2 Materials and methods

### 2.1 Participants

All data is from the Child Mind Institute (CMI) Healthy Brain Network (HBN) Project, which collects neuroimaging and phenotypic data from a diverse sample of participants (Alexander et al., 2017). Neuroimaging data is publicly available for download on the CMI HBN website, while phenotypic data was made available for download via the Longitudinal Online Research and Imaging System (LORIS) upon completion of a Data Usage Agreement (DUA).

#### 2.1.1 Inclusion criteria

We included participants who were ≥6 years of age at the time of data collection, up to the age of 21 (full range 6–21 years). While this is a wide age range, we felt it was important to include a representative sample of the population with sufficient variability in age in order to create a model that was able to reliably detect age-related differences in FC.

**Fig. 1:**
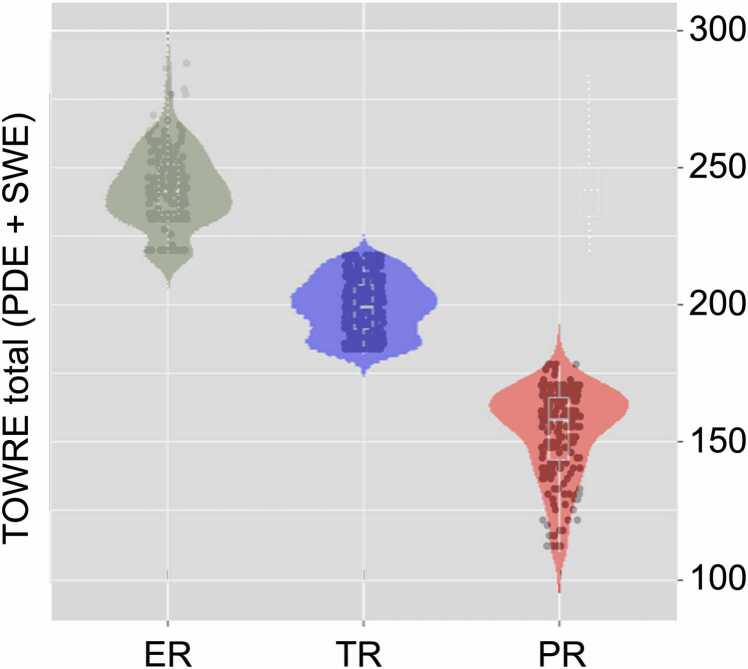
**TOWRE total scores across each group.** Individual dots represent a single participant; violin plots are used to show the distribution of scores within each group.

The Test of Word Reading Efficiency (TOWRE), designed for participants aged 6–24 years, was used for group classification criteria (Torgesen et al., 2012). TOWRE involves two core subtests: Phonemic Decoding Efficiency (PDE) and Sight Word Efficiency (SWE). The researcher presents a participant with a list of either real words (for SWE) or pseudowords (for PDE), which the participant is asked to read aloud one-by-one as quickly as possible. Real word reading is thought to index sight word reading ability, while pseudoword reading is thought to index phonemic decoding skills. The composite Total Word Reading Efficiency (TWRE) index is derived from these two subtests (Fig. 1). Both PDE and SWE are age-standardized with a mean of 100, and the standard scores are averaged to generate the TWRE index. A participant is considered a poor reader (PR) if TWRE < 91, a typical reader (TR) if 91 ≤ TWRE ≤ 109, and an exceptional reader (ER) if TWRE > 109.

#### 2.1.2 Comorbidities

We do not exclude participants based on certain comorbid clinician diagnoses. This was done to avoid the emergent population-level bias in traditional studies which disallow this kind of natural variation. RD has high comorbidity with attention deficit hyperactivity disorder (ADHD) and other specific learning disorders, such as writing (dysgraphia) and mathematics (dyscalculia), as well as internalizing disorders such as depression and anxiety (Hendren et al., 2018). In addition, while RD has often been defined in terms of unexpectedly poor decoding in the absence of intellectual disability (Rutter and Yule, 1975), associations between RD and IQ scores (both verbal and nonverbal) have been reported at the population level, particularly for verbal IQ (van Bergen et al., 2014).

Table 1 contains all demographic and behavioral data on the participants after scan quality-control criteria had been applied. Gender, SES, and handedness were included as covariates/nuisance regressors for the BAG analyses. Nonverbal (performance IQ, or PIQ) was also included as a covariate despite its association with both RD status and SES in our sample, as it may be that any effects of RD status or SES are accounted for in part by group PIQ differences. One-way ANOVAs showing the group differences are in Table 1, and Spearman’s rho for the relationship between SES and PIQ/FSIQ scores are.217 (p < .001) and.322 (p < .001), respectively.

**Table 1:** Demographic & behavioral data.

	**ER** **N = 193**	**TR** **N = 338**	**PR** **N = 211**	**Test statistic**	**Effect size (*****n***^**2**^**)**
**Age**^a^^**¶**^	10.2 (2.9)_a_	10.7 (2.7)_a,b_	11.2 (3.6)_b_	Welch’s F(2407.616) = 4.88 (*p* = .008)**	.015
**Gender (% female)**^**¶**^	29.5	36.7	34.6	***X***^2^(2739) = 2.81 (*p* = .245)	
**Socioeconomic status (SES)**^b^	9.0 (3.0)_a_ [N = 166]	8.5 (3.1)_b_ [N = 280]	6.8 (3.7)_c_ [N = 158]	Kruskal-Wallis H(2) = 37.85 (p < .001)***	.060
**Handedness (% right)**^**¶**^	91.7	86.7	91.0	***X***^2^(2739) = 4.19 (*p* = .123)	
**FSIQ**^a^	114.0 (14.4)_a_	102.6 (12.1)_b_	90.8 (12.6)_c_ [N = 178]	Welch’s F(2380.667) = 136.66 (*p* < .001)***	.298
**PIQ (Block design, Scaled)**	11.6 (3.1)_a_	10.3 (3.2)_b_	8.9 (2.9)_c_ [N = 178]	F(2706) = 36.39 (*p* < .001)***	.093
TOWRE					
**PDE (Scaled)**^a^	118.0 (8.4)_a_	98.4 (7.6)_b_	76.1 (9.3)_c_	Welch’s F(2411.073) = 1139.03 (*p* < .001)***	.778
**SWE (Scaled)**^a^	123.8 (8.6)_a_	100.7 (7.8)_b_	77.5 (10.0)_c_	Welch’s F(2408.330) = 1269.40 (*p* < .001)***	.797

a Levene’s test p < .05; Welch’s statistic and Games-Howell correction used.

b Kruskal-Wallis nonparametric test reported for ordinal variable; Mann-Whitney *U* test for pairwise follow-ups.

Included as covariates for analyses.

Cells with the same subscript indicate non-significant group comparisons at the.05 alpha level. All cells are in the format MEAN (SD) except for gender and handedness, which are given in percentages. IQ was measured using the Wechsler Intelligence Scale for Children – Fifth Edition (WISC-V). Post-hoc tests use Bonferroni correction for multiple comparisons if Levene’s test is not significant. Where data is missing, actual N is given in brackets.

### 2.2 Resting fMRI data acquisition

fMRI data was collected during a movie-watching paradigm: the participants viewed a 10-minute-long clip from the film *Despicable Me* while in-scanner. The clip was presented as part of a longer in-scanner protocol and played at the following time stamp: 01:02:09–01:12:09 (HH:MM:SS). The protocol script was written in PsychoPy2 Experiment Builder v1.83.04 (Peirce, 2007). Details of scanner parameters are given in Supplementary File 1.

### 2.3 Preprocessing

Data was downloaded from the CMI HBN Project neuroimaging data portal at http://fcon_1000.projects.nitrc.org/indi/cmi_healthy_brain_network/ (DOI: 10.1038/sdata.2017.181). Data preprocessing (intensity non-uniformity correction, skull-stripping, and segmentation for anatomical T1 data; susceptibility distortion correction and co-registration for functional data) and spatial registration of anatomical and functional data were performed using fMRIPrep v1.5.8 (Esteban et al., 2019). For detailed information on the fMRIPrep preprocessing pipeline see Supplementary File 1. Regression of confounds from the BOLD signal was performed using the CONN functional connectivity toolbox (Whitfield-Gabrieli and Nieto-Castanon, 2012). Subcortical, white matter, and cerebrospinal fluid signals derived from tCompCor and aCompCor; framewise displacement (FD); and realignment parameters were regressed from the BOLD signal and high-motion frames were removed (‘scrubbing’, described in Power et al., 2012). A bandpass filter of 0.008–0.09 Hz was then applied to the signal, and linear detrending was applied to correct for time-dependent artifactual signal drift.

### 2.4 Quality control & exclusion criteria

Participants were excluded if (1) their functional data did not include a full run of the movie-watching paradigm (e.g., left the scanner early), or (2) their anatomical T1 scan or functional run was determined to be of insufficient quality. For T1 data, MRIQC was used to accept or reject scans based on quality assessed using a 64-feature vector derived from 14 image quality metrics (Esteban et al., 2017). For functional data, the criteria for exclusion was having more than one-third of frames flagged for excess motion (>0.5 mm FD) by fMRIPrep. This left a final sample size of N = 742 participants. A Pearson Chi-squared test found that the rate of exclusion differed between groups: *X*^**2**^(2939) = 9.77 (*p* = .008). Post-hoc tests found that the difference was driven by TRs and PRs: *X*^**2**^(1691) = 9.62 (*p* = .002), with the PRs more likely to be excluded. The difference between TRs/ERs (*X*^**2**^(1653) = 3.21; *p* = .079) and PRs/ERs (*X*^**2**^(1534) = 1.18; *p* = .312), were non-significant.

### 2.5 Regions of interest (ROIs)

This study utilized the Schaefer et al. (2018) 400-ROI seven-network 2 mm Montreal Neurological Institute (MNI) template to segment the brain into 400 distinct ROI time series, with networks based on the functional seven-network template described in Yeo et al. (2011). The seven networks are Visual (Vis), Somatomotor (SomMot), Dorsal Attention (DorsAttn), Salient Ventral Attention (SalVentAttn), Limbic, Control/Frontoparietal (Cont), and the Default Mode (Default/DMN) networks. An ROI-ROI connectivity matrix of Fisher-transformed bivariate correlation coefficients between each pair of time series was generated for each participant. The whole-brain matrix contained 400 *choose* 2 = 79,800 connections.

We next examined a selection of well-known meta-analyses on neuroimaging studies in reading and language (in both children and adults) to generate a set of coordinates in MNI space which could then be mapped to ROIs in the Schaefer parcellation. Papers met the following criteria: (1) included only studies performed with structural MRI/fMRI or PET imaging; (2) focused on group contrasts between participants with and without a reading/language impairment, or a functional contrast between reading/language and control tasks; (3) reported results as coordinates in either Talairach or MNI space; and (4) focused on English-speaking participants or cross-cultural results. Talairach coordinates were converted to MNI using the Lancaster transformation implemented in GingerALE (Eickhoff et al., 2009, Eickhoff et al., 2012, Laird et al., 2010). A total of 26 meta-analyses were examined. Of these, 22 reported a total of 416 unique coordinates. These coordinates were mapped to the ROI in the Schaefer template in which that coordinate was spatially contained (referred to as a given coordinate’s ‘parent ROI’). 49/416 mapped onto non-cortical regions, leaving a total of 367 in-range coordinates. These 367 coordinates loaded onto a total of 152 unique Schaefer ROIs (some unique coordinates were mapped onto the same ROI). The Supplementary Material includes a spreadsheet with citation information for the final meta-analyses (Supplementary File 2), and another spreadsheet containing all extracted coordinates and their associated ROIs (Supplementary File 3).

#### 2.5.1 ROI cutoffs

**Fig. 2:**
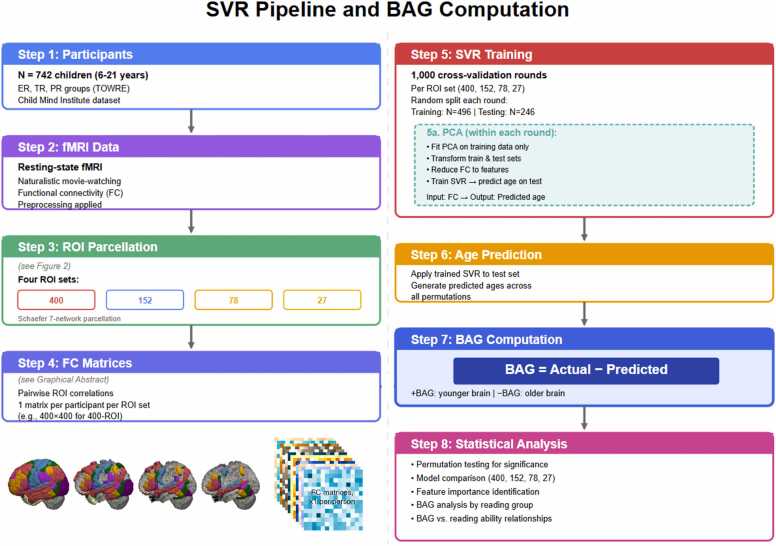
SVR pipeline and BAG computation.

To investigate the specific contribution of reading and language networks to brain-age predictions, four ROI cutoffs were chosen. Cutoffs were based on the total number of coordinates that mapped to a given Schaefer ROI. The greater the number of unique coordinates that map to a given region, the more likely that region is to be part of the core language/reading network and thus relevant to group differences between participants. We created the following sets of ROIs (Fig. 2a-d): 400 ROIs (whole-brain/no cutoff); 152 ROIs (≥1 coordinates mapped to region); 78 ROIs (≥2 coordinates); and 27 ROIs (≥4 coordinates). For the subsequent analyses, pairwise ROI-ROI connections were derived only between included ROIs. As the ROI cutoff increases, the data used to train the models becomes more concentrated in reading network regions.

### 2.6 Model training, cross-validation (CV), and testing

Prior to SVR model training, PCA was applied to all FC data in the training set to reduce the number of features while preserving data variance. PCA converts the feature set into a smaller number of uncorrelated principal components (PCs), each a linear combination of correlated original features (Leonardi et al., 2013, Amico and Goñi, 2018). The linear transformation is computed from the top eigenvectors of the covariance matrix, ranking components by explained variance (Khosla et al., 2019). PCA was performed for each set of ROIs using Python’s Scikit-learn, retaining 95% of the original variance.

Each SVR model was built using Scikit-learn. A linear kernel was chosen to ease model interpretability, and hyperparameter *ε* was set to 0.1 such that the model received no penalty in the training loss function for data points predicted within 0.1 years of the true age value. Pre-PCA features were all pairwise ROI-ROI Fisher’s Z-transformed bivariate correlation coefficients between ROIs. The final set of training features consisted only of the PCs which remained after running PCA when preserving 95% of the variance. Reading ability was not used to train the model. Training labels were floating-point numbers describing chronological age for all participants.

For each model, 1000 training permutations were done, each with a unique split for training/CV and hold-out testing data. For each permutation data was split into the training/CV and test sets using a 2–1 train-test ratio (N = 496/N = 246) prior to any model training or CV. Within each permutation, 5-fold CV was done *on the training set only* using Scikit-learn’s built-in method. The splitting procedure was performed for all three groups (PR, TR, ER) separately to ensure that each group was represented proportionally in both the training and testing sets. For each group a random 2–1 train-test split was performed. The three separate training groups were then combined to form a single training group where each group was represented proportionally; the same was done for the testing set. PCA was then performed on the training data only, to ensure that the test set was withheld from all models throughout training and CV. The resulting PCA model was used to transform the training data prior to training/CV, and the same model was used to transform the test set prior to testing. This means that there are slight differences in the PCA models at each permutation, due to the variation in the training dataset to which each PCA model was fit. However, the number of components retained across permutations was largely consistent. On average, the 400-ROI model retained 426 components, the 152-ROI model retained 394, the 78-ROI model retained 339, and the 27-ROI model retained 153 (rounded to the nearest whole number).

Finally, permutation testing with 1000 iterations was performed in order to assess CV significance of each model. Model performance was assessed using mean absolute error (MAE). For each iteration, the CV process was the same as for the true model, except that training age labels were shuffled prior to training. Each iteration produces a value for CV accuracy, resulting in a null distribution to test the real models’ CV accuracy against. Model stability was assessed by calculating the average coefficient of variation (CoV, SD / mean) of MAE for test set predictions across all permutations.

### 2.7 Analyses

#### 2.7.1 Between-model differences in age prediction (whole-brain vs. reduced-ROI)

We compared prediction accuracy between the whole-brain and reduced-ROI reading network models. Predictions were averaged for each participant across all permutations. All models were compared on their ability to correctly predict age across all participants*,* regardless of decoding ability. Models were compared by extracting subject-level absolute errors from the hold-out test set across all permutations, averaging these values within participants (to generate subject-level test MAE for all participants, N = 742), and then performing a Wilcoxon signed-rank test on these participant-level MAE values for all model pairs. Wilcoxon signed-rank tests were chosen due to violations of normality in the error distribution. We hypothesized that there would be a positive relationship between model performance (minimized prediction error) and the number of ROI connections (prior to PCA) used to train the model. Therefore, models trained with whole-brain data would more accurately predict the chronological age of the participants, collapsed across all three reading groups.

#### 2.7.2 Identification of the most important ROIs / connections (whole-brain model, exploratory)

For each training/testing permutation, the PCA coefficient matrix (N x M; N = number of PCs, M = number of ROI-ROI connections) and SVR model coefficient array (1 x N) were matrix-multiplied to get an average coefficient for each original ROI-ROI connection (1 x M). These 1 x M arrays were averaged across all permutations to get a final average coefficient for each ROI-ROI connection. The coefficients were then ranked and sorted by absolute value, and the top features which met the threshold for significance (*p* < .05) using permutation testing were selected for further examination. Permutation testing was performed by randomly permuting the coefficient values for all features 2500 times, then using these 2500 permutations as a null distribution of coefficients for each feature. The absolute value of a coefficient represents that feature’s importance in model predictions. The top features were then decomposed into their component ROIs, and these ROIs were then pooled together to determine which occurred most frequently in the highest-ranked features. Each ROI’s assigned brain network from the Schaefer parcellation was also extracted. Permutation testing was then used to test whether the most frequent ROIs and networks within the top features were overrepresented compared to random chance; this was done by randomly selecting (with replacement) the same number of ROIs as were present in the set of significant features from the full set of ROIs in the Schaefer parcellation 2500 times. These randomly-chosen ROIs were also assigned to their respective networks. This gave us 2500 sets of null ROIs and their networks, each of which should result in a sampling distribution that is representative of the spatial extent of each network. We use these samplings as the null frequency distribution for each ROI and network. For each of the 400 ROIs tested, we used a strict α value of.0001 to negate the impact of Type I error rates and to result in a manageable/interpretable number of the ‘most important’ ROIs.

#### 2.7.3 Between-group differences in the brain-age gap (BAG)

We calculated the BAG for each participant’s average age prediction across all testing permutations, which is quantified as True Age – Predicted Age. Positive BAG values indicate that the model *under*estimates the true age of the subject, while negative values indicate *over*estimation of age. We compared group differences in overall mean BAG and further examined whether this bias interacted with model type (whole-brain vs. reduced-ROI models). We expected that there would be a difference in the BAG based on reading ability (main effect of Group); and that these effects would be preferentially observed in the reduced-ROI models rather than the whole-brain model.

## 3 Results

### 3.1 Model significance and between-model differences in age prediction

All raw model predictions were first converted to z-scores, multiplied by the standard deviation (SD) of the sample’s age, and added to the sample’s mean age to ensure predictions were on the same scale as the true age. This was done to ensure that error metrics were not overly inflated while preserving the model’s relative age predictions across participants. Error metrics for all models (for both CV and hold-out test set performance), a measure of model stability (CoV), and permutation testing results are shown in Table 2. For model stability, low CoV values (<1 or < 100%) indicate higher clustering of values around the mean across permutations (greater stability). A value of exactly 1 or 100% indicates that the mean and standard deviation of the distribution are equal. MAEs were slightly lower in the hold-out test set compared to CV, suggesting that all models were able to generalize well to unseen data when compared to their CV performance. All models were also relatively stable, with low CoV values indicating that MAEs across permutations are closely clustered around the mean (low variability).

**Table 2:** Error metrics and stability for each SVR model.

	400-ROI	152-ROI	78-ROI	27-ROI
MAE (test set)	2.04	2.17	2.30	2.47
MAE (CV)	2.85	3.03	3.22	2.82
*p-*value	< .001***	.001**	.093	< .001***
Stability (CoV, %)	4.70	4.71	4.90	5.01

Units for MAE are in years. Lower error indicates better performance. MAEs for CV are averaged across folds. P-values are derived from CV permutation testing with 1000 null models (shuffled age labels) as described in the Materials and Methods. CoV = Coefficient of variation, a measure of model stability defined as the % ratio between the standard deviation and the mean for MAEs across all permutations. Values for CoV are given in %.

**Fig. 3:**
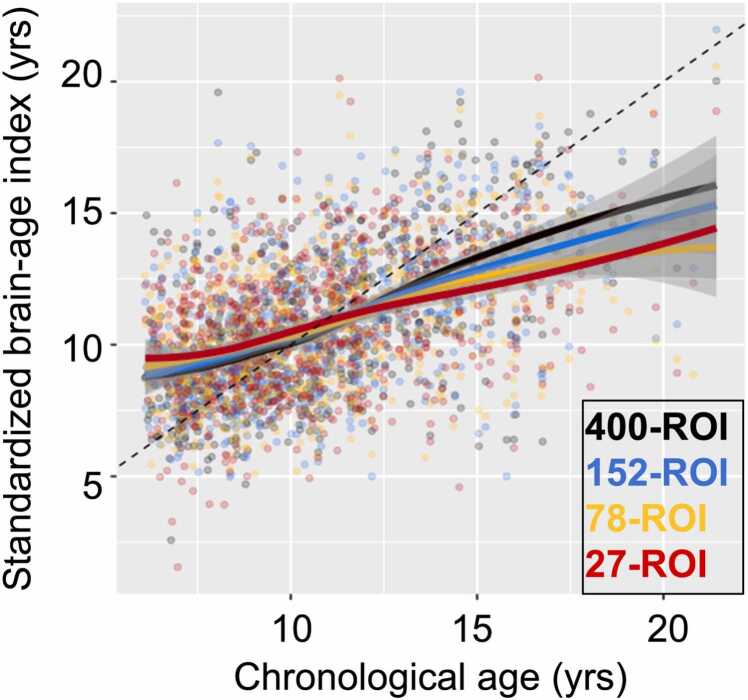
**True age (x-axis) vs. standardized predicted age (y-axis) for all models.** Dotted reference line is at *y = x*. Lines of best fit for individual models are smoothed.

The Shapiro-Wilk tests of normality (implemented in scipy v1.14.1) for averaged subject-level MAE (derived from the hold-out test sets across all permutations) were significant in all models. Due to the violation of normality, paired tests of subject-level MAE utilized the Wilcoxon’s signed-rank test rather than a traditional paired *t*-test. Results showed that (1) the whole-brain model minimized prediction error (quantified by total MAE, averaged across all folds) relative to the other models (Tables 2), and (2) the whole-brain model significantly outperformed the other three models in terms of subject-level MAE (Table 3). Best-fit regression lines for all models and corresponding *R*^*2*^ are shown in Fig. 3.

**Table 3:** Pairwise model comparison of subject-level MAE (from hold-out test set), N = 742.

Model comparison	Statistic	Effect size	*p*
400-ROI vs. 152-ROI	W = 99421	.14	< .001***
400-ROI vs. 78-ROI	W = 83908	.27	< .001***
400-ROI vs. 27-ROI	W = 82529	.35	< .001***
152-ROI vs. 78-ROI	W = 99805	.13	< .001***
152-ROI vs. 27-ROI	W = 100600	.21	< .001***
78-ROI vs. 27-ROI	W = 120524	.08	.003**

Paired tests were conducted using subject-level MAEs (averaged within subject) derived from the hold-out test set errors across all permutations.

All tests are Wilcoxon’s signed-rank due to violations of the normality assumption.

Effect size is given as Cohen’s d.

*p < .05

** p < .01,

*** p < .001

### 3.2 Identification of the most important ROIs / connections (whole-brain model)

#### 3.2.1 Connectivity feature importance

**Fig. 4:**
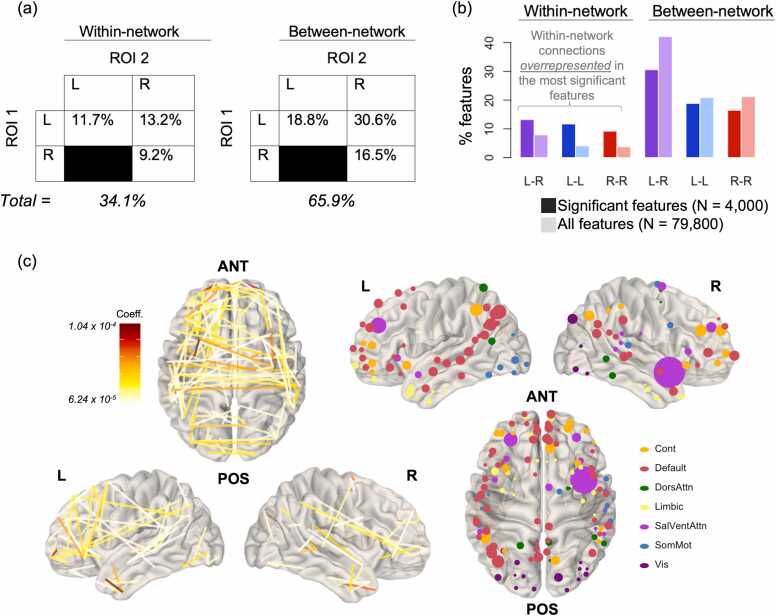
**Significant connections and their hemispheric origins.** (a) The N = 4000 significant connections were predominantly between-network (65.9%), interhemispheric (43.8%), or both (30.6%). (b) Comparison of the percentages in (a) to those from the full set of N = 79,800 connections shows that within-network connections are overrepresented among significant features, regardless of hemispheric identity. (c) Connections with coefficient magnitudes > 2.5 SDs above the mean (left) and their associated ROIs (right), with ROI size weighted by relative frequency within the significant connections. ANT = Anterior, POS = Posterior.

Results from permutation testing showed that only 4000 features (5.01% of the total feature set) met our criteria for significance in the whole-brain model. Most were between-network (65.9%) rather than within-network (34.1%) (Fig. 4a). A plurality of significant connections was interhemispheric (43.8%), followed by left-hemisphere connections (30.5%) and right-hemisphere connections (25.7%). The interhemispheric connections were predominantly between-network, at a rate of 69.9%. However, comparing these rates to those of the entire whole-brain connectivity matrix shows that the within-network connections were actually *overrepresented* in the set of significant features, while between-network connections were underrepresented, regardless of hemispheric identity (Fig. 4b). We also visualized those connections with absolute coefficient values > 2.5 SDs above the mean and overlaid them onto an MNI-template brain (Fig. 4c, left), along with their component ROIs (Fig. 4c, right).

#### 3.2.2 ROI importance

**Fig. 5:**
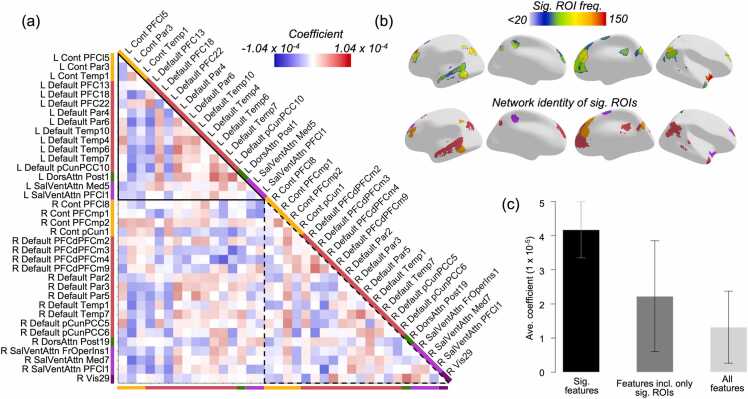
**Top ROIs in the whole-brain model.** (a) Feature coefficients for each pair of significant ROIs. Colored bars on each side of the triangle reflect each ROI’s assigned network in the Schaefer parcellation. (b) Top row: color represents the frequency of occurrence for each significant ROI in the 4000 significant connections. Bottom row: color represents the assigned network for each significant ROI. (c) Features including only significant ROIs (middle bar) have higher average coefficient values than all features together (right), but lower average coefficient values than the 4000 significant features (left).

We found that 36 ROIs in the Schaefer parcellation met our strict permutation-testing criteria for being overrepresented in the set of significant connections (Fig. 5a, b). The median frequency of all ROIs in the significant features was 18 (SD = 13.73). Among the 36 significant ROIs, the median frequency within the significant features was 45 (SD = 19.09, range 36–150). These regions were primarily drawn from temporal, frontal, and parietal regions of cortex, with little representation from occipital regions. The set of features associated only with these ROIs (36 choose 2 = 630 connections) had a larger average absolute feature coefficient than the full set of features (Fig. 5c). The region with the single greatest frequency of occurrence within the significant features was the frontal operculum/insula (SalVentAttn), which occurred in 3.75% of significant features.

#### 3.2.3 Network representation

We used the same set of permutations generated for our ROI testing procedure to determine if the significant ROIs/connections came predominantly from specific brain networks. We used the ROI-based permutations due to the fact that certain networks cover a larger spatial area and consist of greater numbers of individual ROIs than others, meaning that their representation in a randomly-selected sample of ROIs will already be skewed.

**Fig. 6:**
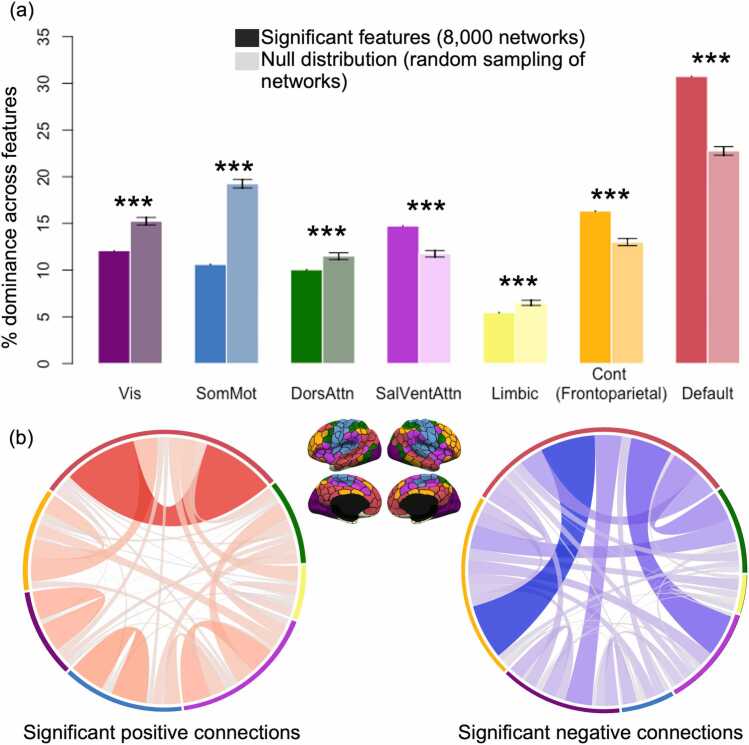
**Network representation among the most significant features in the whole-brain model.** (a) Darker bars represent a network’s relative dominance in the set of 8000 ROIs derived from the 4000 significant connections. Lighter bars represent a network’s average relative dominance across all 2500 permutations for randomly-chosen ROIs. Error bars are given in SD. ***p < .001. (b) Connectivity patterns differed based on the sign of coefficients.

Results showed that three networks were overrepresented in the significant features (the SalVentAttn, Control, and Default networks), while the other four were underrepresented (the Visual, SomMot, DorsAttn, and Limbic networks) (Fig. 6a). Both positive and negative coefficients were dominated by connections involving the DMN, although the nature of those connections differed: the positive connections were dominated by within-network connections of the DMN and other networks, while negative ones were dominated by connectivity between the DMN and the Control/SalVentAttn networks (Fig. 6b).

### 3.3 Between-group differences in the BAG

Pairwise Pearson’s correlations between all model BAGs were high (r = .658–892, all *p*s < .001), as were pairwise correlations between predicted ages for all models (r = .600–.893, all *p*s < .001).

**Fig. 7:**
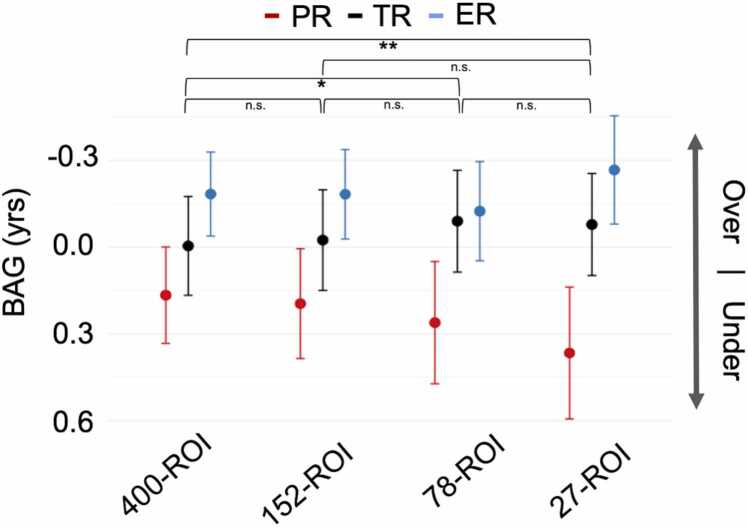
**Mean BAG values for all models, separated by reading group.** Positive values indicate underestimation of true age, while negative values indicate overestimation. Line and dot color indicates reading group. Error bars are given in standard error. Significance indicates a pairwise Model difference in BAG (collapsed across group). ***p < .001, **p < .01, **p < .05, n.s. = not significant.

We ran a repeated measures analysis of covariance (RM-ANCOVA) on BAG values, with Model (400-ROI, 152-ROI, 27-ROI) as a within-subjects effect, Group (ER vs. TR vs. PR) as a between-subjects effect, and true age as a covariate, with interaction effects included. The 78-ROI model was excluded from the repeated measures variable due to having a CV significance of p > .05 (Table 2). Other covariates of no interest were gender, SES, PIQ, and handedness. In the model without nuisance covariates (but still including age, due to the strong correlation between true age and BAG values: r = .515–.654; all *p*s < .001) there was a significant effect of Group, F(2,736) = 14.78, *p* < .001, partial η^2^ = .039. However, there was no Model x Group interaction (*p* = .359, Greenhouse-Geisser corrected for violations of sphericity). When nuisance covariates were included, the main effect of Group, F(2,578) = 10.12 (p < .001, partial η^2^ =.034) and lack of Model x Group interaction (p = . 429, Greenhouse-Geisser corrected) were preserved. Post-hoc separate RM-ANCOVAs (including all covariates) in each group confirmed a main effect of Model in the PRs (F(1.528,207.805) = 4.27, p = .024, Greenhouse-Geisser corrected) and ERs (F(1.391,222.547, Greenhouse-Geisser corrected) = 4.92, p = .017) but not the TRs (p = .064, Greenhouse-Geisser corrected). Post-hoc comparisons with Bonferroni correction did not result in significant pairwise comparisons for either PRs (all ps >.548) or ERs (all ps >.335). Examination of descriptive plots implied that these effects were driven primarily by a trend towards a more negative BAG in the ERs (overestimation of age) and a trend towards a more positive BAG in the PRs (underestimation of age) (Fig. 7).

Due to the presence of a Group x Age interaction in the main model without additional covariates (aside from age), F(2,736) = 15.29 (p = <.001, partial η^2^ =.040), we ran a series of post-hoc analyses in which we included age bins as an additional between-subjects factor, to examine the nature of the group differences in BAG for each model (400-ROI, 152-ROI, and 27-ROI) in younger, middle, and older participants. Bins were as follows: Bin 1 = 6–8 years (N = 153), Bin 2 = 8–9.5 years (N = 152), Bin 3 = 9.5–11 years (N = 129), Bin 4 = 11–13 years (N = 136), Bin 5 = 13–15 years (N = 93), and Bin 6 = 15–21 years (N = 79). All post-hoc models controlled for chronological age, as in the main analysis. Results from the 400-ROI model showed that for Bin 1, ERs had a larger BAG than TRs (Cohen’s d = −.754, p = .009, Bonferroni-corrected); however, this effect had reversed by Bin 6, wherein ERs had a smaller BAG than TRs (Cohen’s d = 1.179, p = .023, Bonferroni-corrected). There were no other significant pairwise comparisons.

**Fig. 8:**
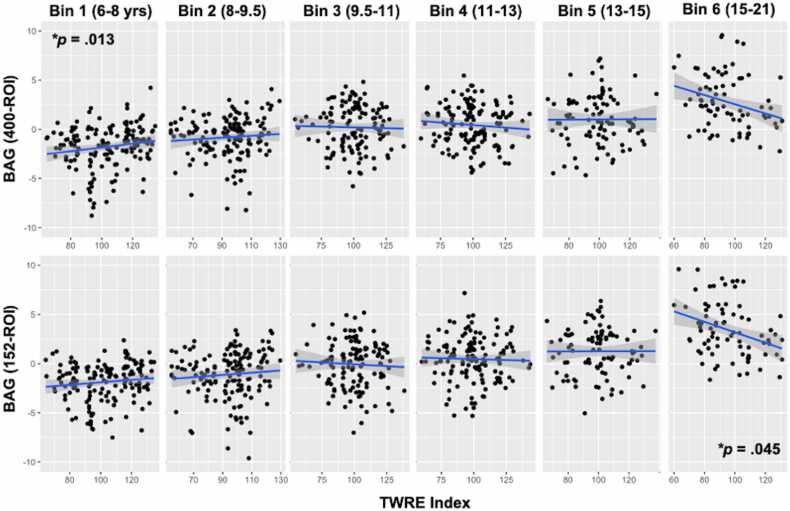
**Relationship between TWRE Index and BAG values in separate age bins for the 400-ROI and 152-ROI models**. The positive effect of TWRE diminished from the first to last age bin in both the 400- and 152-ROI models. *p < .05.

Next, to determine whether there may be continuous effects of reading ability on BAG, we performed a post-hoc linear regression analysis on each model’s BAG separately using both age and the TWRE index as continuous predictors (excluding RD status). All covariates were included. A TWRE x Age interaction effect was also included, as in the main RM-ANCOVA. All three linear models were significant, F(7,580) > 25.89 (all ps <.001). However, only the 400-ROI and 152-ROI models had a significant positive main effect of TWRE index (400-ROI: t = 2.58, β =.387, p = .010; 152-ROI: t = 2.18, β =.313, p = .030) and a significant TWRE x Age interaction (400-ROI: t = -2.54, β = −.686, p = .012; 152-ROI: t = -2.27, β = −.587, p = .024). The effect of TWRE index and the TWRE x Age interaction in the 27-ROI model were n.s. (p = .095, p = .087). However, contrary to expectations, the main effect of TWRE in each case was positive. Follow-up analyses of BAG in each age bin (due to the presence of a TWRE x Age interaction in all models) demonstrated that, in the 400-ROI model, the positive TWRE-BAG relationship existed only in Bin 1 (t = 2.53, β =.256, p = .013) but diminished at subsequent age bins (Fig. 8). For the 152-ROI model, in contrast, the TWRE-BAG relationship also decreased across age bins, but in a different way: effects of TWRE were n.s. at younger age bins and a significant negative relationship emerged only in the oldest cohort, Bin 6 (t = -2.07, β = −.325, p = .045).

Finally, we performed an additional supplementary analysis in which we adjusted the group cutoff criteria to determine if the choice of TWRE index cutoff could have impacted these results. However, this analysis did not impact the significance of the interaction effects (Supplementary File 4).

## 4 Discussion

In summary, we successfully trained a model to predict age based on FC data and showed bias effects in model predictions based on reading status. Connections involving frontoparietal control regions and the DMN were the most heavily weighted in predicting age. Contrary to our hypothesis, however, the 27-ROI model did not show a greater effect of Group on BAG compared to the other models. Rather, all models showed a group bias in the BAG, but follow-up pairwise comparisons did not support the hypothesis that the PRs’ ages are consistently underestimated relative to the other two groups. Rather, only for the TR-ER comparison were the group discrepancies in BAG present in the expected direction at older ages, and only in the 400-ROI model.

The exploratory results suggest that regions and connections with the most predictive value come from the DMN and the frontoparietal control networks. Interconnectivity within regions in the DMN are known to strengthen across childhood development (Buckner et al., 2008, Fair et al., 2008) and reflect advancement in a variety of cognitive processes, including reading (Bailey et al., 2018). While the frontoparietal control network is thought to index cognitive control and executive functioning – both key areas of cognitive development from childhood to adulthood – a role for frontoparietal regions has long been posited in reading, particularly RD (Hoeft et al., 2006, Hoeft et al., 2011, Schwarze et al., 2023, Mascheretti et al., 2024). A slim majority of the densest reading-related regions from our literature review in this study were identified as belonging to frontoparietal or cingulo-opercular control networks, and the DMN (14/27 ROIs). Taken together, the overlap between regions most implicated in reading meta-analyses and those identified as contributing the most to age predictions suggests a strong link between reading maturity and age-related brain development (Vogel et al., 2013). There are two perspectives here to consider: the first is that the DMN and frontoparietal networks either have a strong interaction with primary reading-related areas or are themselves interleaved to some extent within the reading network; the second is that the DMN and frontoparietal networks support domain-general functions that subserve reading development in a non-specific way.

Regarding how these networks map onto reading processes, the DMN contains regions long implicated in reading-related activation, such as the temporal lobes and angular gyrus (Martin et al., 2015, Bailey et al., 2018); both of which overlapped with significant ROIs in the whole-brain model. This would seem to suggest that the DMN follows the first perspective. On the other hand, the frontoparietal network is a key component of the dorsal visual stream, and disturbances in connectivity could interfere with both attentional and perceptual processes, which have long been thought to be atypical in RD (Mascheretti et al., 2024). This would seem to suggest that frontoparietal network contributions may fall within the second framework. In any case, the brain networks subserving reading undergo major development in their connectivity patterns from early childhood to late adolescence, resulting in their relative dominance in predicting age in this sample; as such, it is likely that this degree of overlap may not be present when studying development from late adolescence into adulthood (when reading development plateaus).

This period of childhood development is characterized by relative strengthening of co-activation between regions within the same functional network (particularly in the DMN and frontoparietal control networks). Our results also confirm prior findings that show anticorrelated connectivity between the control network and DMN from childhood to late adolescence, given that our most negative significant connections were disproportionately connections between the control network and the DMN (Fig. 6b; see also DeSerisy et al., 2021). These results are reflective of these networks’ status as ‘task-active’ vs. ‘task-inactive’, respectively. Altogether, we have observed a relative strengthening of local connections throughout early childhood and adolescence, with a disproportionate emphasis on connectivity within functional networks, although between-network connectivity still plays a dominant role during development in this stage of life.

The whole-brain model was a better overall predictor of age than the other three reduced-ROI models based on MAE (see Table 2). Similarly, the pairwise tests between models indicated that the whole-brain model significantly and reliably outperformed all other models, in line with our expectations (Table 3). However, it is worth noting that the level of variance in true age explained between the whole-brain model (R^2^ =.343) and the most reduced-ROI model with 27 ROIs (R^2^ =.155) decreased only by approximately half, despite the models’ features being constructed from vastly different amounts of data. The total number of connections used to construct features for the 27-ROI model (351 pairwise connections) was < .005% of the amount used for the whole-brain model (79,800 connections). Yet, this model was still able to capture a significant amount of variance in age based on connectivity between a highly restricted set of brain regions and still showed a main Group effect in BAG. This suggests that while training with widely-diffused whole-brain data will result in more accurate age estimates, accuracy is not linearly proportional to the total number of ROIs or connections used to generate features. Markers of age, in addition to markers of reading ability, are diffusely represented in connectivity patterns across the brain.

We also predicted that there would be a larger group bias in our model’s age predictions when it was trained to predict age using FC data from reading network ROIs drawn preferentially from left-hemisphere reading and language regions rather than diffuse whole-brain connectivity data. Contrary to our expectations, there was no Model x Group interaction that showed greater bias in the 152- or 27-ROI models. While visual examination of each group separately showed the expectation direction of effect (the ages of PRs were underestimated, and the ages of ERs were overestimated, while the TRs had an average BAG closer to 0), post-hoc pairwise comparisons involving the PRs were not significant in any of the models. In fact, only in the 400-ROI model did we see a pairwise difference in BAG. In early childhood ERs actually had their ages *underestimated* relative to TRs, but by late adolescence the ERs showed a reversal of this effect and were instead being predicted as *older* than TRs (even when controlling for true age). There are a few considerations here: (1) the lack of clear pairwise differences involving the PRs suggests that the observed differences are subject to large individual variation and may not be reliable or may be better detected using an alternative task paradigm; (2) the dynamic observed for ERs may indicate that these children *do* follow an atypical developmental trajectory, but that it is nonlinear. Future studies should take this possibility into account when examining developmental dynamics of reading development. Furthermore, when reading ability was modeled as a continuous (TWRE index) rather than group-based measure, its relationship to BAG was moderated by age. This suggests that other developmental factors may moderate the relationship between TWRE and the BAG. One possibility is that the results from Bin 1 were skewed by the presence of younger precocious readers (ERs), while the results from Bin 6 are consistent with the expectation that those with faster developmental trajectories for their true age (older-appearing brains) are better readers.

The PRs and ERs also had a lower absolute BAG and higher R^2^ values than the TRs, meaning that while the average error in these groups was skewed positive or negative, the magnitude of the error was lower compared to the TRs. This may be interpreted as the trained models being better fit (but with greater bias) to data from the PRs and ERs or, alternatively, the TR group having a higher amount of variability within the population, which the models struggled to capture (Supplementary File 4). A possible follow-up to these results may be to examine whether the same functional connections identified as driving the model’s predictions in our exploratory analysis also differ between these reading groups, allowing for more reliable and direct group comparisons.

## 5 Limitations

There are several limitations to the current study. First, the SVR model coefficients are agnostic towards the initial sign or magnitude of the correlation between two ROIs. Bearing this in mind, the developmental trajectories of highly-weighted connections are only explored in terms of either increasing or decreasing with age, rather than more precise statements about their initial state and developmental dynamics (which may be nonlinear). Another point of criticism is that we have trained the SVR model to predict age using a representative split from the PR, TR, and ER populations. Therefore, the model was trained to predict the age of poor (and exceptional) readers as much as it was trained to predict the age of controls. An alternative approach that uses the latter method of training only with controls may produce clearer bias effects.

A more robust approach may also involve matching the groups more strictly on various criteria, including demographic variables. One confound, particularly for the BAG analyses, is that there are slight differences in age between reading groups. While we tried to control for this in our analyses by including true age as a covariate, it is unclear whether having groups better matched on demographic variables would have produced the same results. Furthermore, we do not assess the model’s ability to predict reading skills directly. A follow-up study that instead assesses the model’s ability to predict continuous reading scores or diagnostic status rather than age would be more enlightening about which functional networks in the brain underlie reading ability uniquely.

An additional consideration is that the topological overlap between the highest-weighted regions and reading/language regions may be indicative of the engagement of language regions by the movie watched during data collection, rather than intrinsic activity. Therefore, our model would have been predicting age based on the maturity of brain network responses to linguistic processing, in which case we would expect these regions to have high overlap with our *a priori* ROIs. It is not obvious (or perhaps likely) that true intrinsic functional connectivity would have produced the same pattern of results, and a model trained with functional data from a different battery of tasks (or true resting-state) may even be a better predictor of age. It would be particularly interesting to replicate these results using a naturalistic in-scanner reading paradigm.

Finally, regarding our choice of ROIs, the decision to perform a literature review of meta-analyses means that there is some degree of study duplication in our ROI results. Researchers who wish to use a similar methodology in the future may consider performing their own meta-analysis or extracting coordinates from a single, comprehensive meta-analysis to avoid this issue. Additional details on the extent of study overlap for our literature review are presented in Supplementary File 5. One effect of this overlap may be that certain ROIs – whose significance is reported in highly-cited papers and are therefore more likely to appear across meta-analyses – may have their apparent importance to the reading network inflated. This would primarily affect the results of the models trained with a smaller number of ROIs because we use the raw number of occurrences across meta-analyses for ROI inclusion, without controlling for duplication. While potentially problematic, we do not expect that this would have significantly impacted the results, given our final reading networks (visualized in Fig. 2) are consistent with previous literature on the neural correlates of reading and language. Nonetheless, a similar study may consider attempting to control for study duplication when ranking ROIs by their number of occurrences across meta-analyses: one way this may be done is by de-weighting ROIs whose reference meta-analyses contain a higher degree of study overlap.

## 6 Conclusion

Integrating machine learning with neuroimaging provides promising avenues for advancing diagnostic and intervention strategies for children with neurodevelopmental and learning disorders. This study illustrates that models trained on FC data are able to reliably predict participants’ true age, although there is some ambiguity as to whether group bias in age predictions are statistically reliable or linear. Future research could focus on refining this model and/or predicting reading skills as opposed to age in order to identify ‘age-agnostic’ brain networks underlying reading development.

## Ethics approval statement

Neuroimaging and behavioral data from the Child Mind Institute Healthy Brain Network Project are publicly available upon completion of a Data Usage Agreement (DUA). The DUA was completed by the authors prior to data access. Data were provided in a de-identified and anonymized format. Therefore, none of the authors were given access to any information which could be used to re-identify the participants. Additional participant consent or involvement was thus not required and, consistent with Institutional Review Board (IRB) policy at the University of Connecticut, further ethical approval was not sought from the University of Connecticut’s IRB.

## Funding sources

Author OHML was supported by the following National Institutes of Health (10.13039/100000002NIH) grant(s): T32DC017703 and F31HD107944; and the National Science Foundation (10.13039/100000001NSF) grant NRT-UtB1735225. Author FH was supported by the following grants: NSF NRT-2152202, NIH R01HD094834, NIH R01HD096261, NIH U24AT011281, USDA/NIFA 2023–70440–40144, NSF MCA-2120888, NIH R01DC013064, and DoE GAANN P200A210098.

## CRediT authorship contribution statement

**Brianna Kinnie:** Writing – review & editing, Data curation. **Shaan Kamal:** Software, Methodology, Data curation, Conceptualization. **Jie Luo:** Writing – review & editing, Writing – original draft. **Oliver H.M. Lasnick:** Writing – original draft, Visualization, Software, Methodology, Funding acquisition, Formal analysis, Data curation. **Spencer Low:** Data curation. **Natasza Marrouch:** Supervision, Conceptualization. **Fumiko Hoeft:** Writing – review & editing, Supervision, Project administration, Funding acquisition, Conceptualization.

## Declaration of Competing Interest

The authors declare that no competing interests exist.

## Data Availability

Data will be made available on request.

## Glossary

Developmental dyslexia / reading disorder (RD): A specific learning disorder affecting reading ability in the absence of any other explanatory condition such as intellectual disability or visual impairment
Support vector regression (SVR): A supervised machine learning technique which predicts continuous outcomes (such as chronological age) rather than classifying each observation; finds the best-fit function within a defined error margin
Principal component analysis (PCA): A dimensionality reduction technique that transforms a high-dimensional dataset with many features per observation into a reduced set of ‘principal components’ for each observation; each component is a linear combination of several original (correlated) features, and the final set of components are all orthogonal (uncorrelated) to one another
Brain-age gap (BAG): The difference, given in units of time, between a participant’s true chronological age and a predictive model’s estimated age for that participant based on brain data (Actual – Predicted)
