# Supplementary material for: Modeling differences in neurodevelopmental maturity of the reading network using support vector regression on functional connectivity data
Source: Dev Cogn Neurosci. 2026 Mar 25;79:101716. doi: 10.1016/j.dcn.2026.101716 (PMC13081699; doi:10.1016/j.dcn.2026.101716)
Supplement: Supplementary file 5 — Supplementary material [file mmc5.docx]

**Statistical Analysis of the Overlap Between Meta-Analyses (Literature Review)**

Our methodology for defining the various reduced-ROI reading/language networks involved compiling the results of 22 meta-analyses which examined either (1) group contrasts between participants with and without a reading/language impairment or (2) a functional contrast between reading/language and control tasks. However, given the similar nature of these papers’ topics, it is implausible that there would not be some degree of overlap in the lists of included studies across all meta-analyses. We would like to emphasize that the goal of our approach was to identify those centroids considered to be the most “replicable” across meta-analyses examining a diversity of reading- and language-related task activations. Nonetheless, readers should be informed as to the degree of the overlap in the source material. Therefore, we extracted all included original studies from each of the 22 included meta-analyses that compiled our literature review and present the results here for the readers’ convenience. The average number of papers included in each meta-analyses was M = 27.18 (SD = 23.45), with a median of 19.5.

**Frequency of all original included studies.** Across all meta-analyses, there were a total of N = 346 unique original papers from which coordinates were derived. The average frequency of occurrence (the number of unique meta-analyses in which that study is included) across all papers was M = 1.73 (SD = 1.31). Table S2 illustrates the proportion of studies included only 1-3 times or greater across all meta-analyses.

**Table S2. Study frequency across all meta-analyses.**

|  | **Number of papers** | **% of total** |
| --- | --- | --- |
| Included x1 (no duplicates) | 219 | 63.3% |
| Included x2 | 72 | 20.8% |
| Included x3 | 23 | 6.6% |
| Included ≥4 | 32 | 9.3% |

**Pairwise citation overlap across all meta-analyses.** Additionally, we examined the extent of the overlap in citations between any given pair of meta-analyses in our literature review, to provide a rough estimate of how similar we would expect the overall meta-analytic results to be between any two meta-analyses. Those with greater citation overlap would likely produce highly similar and thus “duplicated” results, inflating the apparent importance of certain ROIs.

For two unique meta-analyses in our sample, A and B, we define the *pairwise citation similarity* for “A” relative to “B” as the percentage of A’s citations that are also included in B. Note that the pairwise citation similarity for “B” relative to “A” is not necessarily the same, as B may have many more included studies than A, which would drive down the B-A citation similarity score. Thus, each of our 22 meta-analyses has a set of 21 pairwise citation similarity values. Across all meta-analyses, the average pairwise citation similarity (interpreted as the average % overlap between that paper and all the others) was 9.75% (SD = 5.86%). The median pairwise citation similarity was 8.43%.
