# Supplementary material for: Modeling differences in neurodevelopmental maturity of the reading network using support vector regression on functional connectivity data
Source: Dev Cogn Neurosci. 2026 Mar 25;79:101716. doi: 10.1016/j.dcn.2026.101716 (PMC13081699; doi:10.1016/j.dcn.2026.101716)
Supplement: Supplementary file 6 — Supplementary material [file mmc6.docx]

**Supplementary File 1. Additional preprocessing details.**

**Supplementary File 2. Meta-analyses used to extract reading-related regions.**

**Supplementary File 3. Coordinate data, regional information, and metadata for all extracted coordinates from meta-analyses.**

**Supplementary File 4. Supplementary results, including analysis of demographic covariates and follow-up BAG and connectivity analyses.**

**Supplementary File 5. Statistical analysis of the overlap in literature between meta-analyses used for this study.**
